# Implementation of a clinical long-term follow-up database for adult childhood cancer survivors in Germany: a feasibility study at two specialised late effects clinics

**DOI:** 10.1007/s00432-023-05145-8

**Published:** 2023-07-18

**Authors:** Madelaine Sleimann, Magdalena Balcerek, Chirine Cytera, Franziska Richter, Anja Borgmann-Staudt, Bernhard Wörmann, Lea Louisa Kronziel, Gabriele Calaminus, Ann-Kristin Kock-Schoppenhauer, Desiree Grabow, Katja Baust, Anke Neumann, Thorsten Langer, Judith Gebauer

**Affiliations:** 1 Medizinische Klinik 1, Abteilung für Endokrinologie, Diabetologie und Stoffwechselmedizin, Universitätsklinikum Schleswig-Holstein, Campus Lübeck, Ratzeburger Allee 160, 23538 Lübeck, Germany; 2 Department of Paediatric Oncology and Haematology, Charité-Universitätsmedizin Berlin, Corporate Member of Freie Universität Berlin, Humboldt-Universität zu Berlin, Berlin Institute of Health, Campus Virchow-Klinikum, Augustenburger Platz 1, Mittelallee 6A, 13353 Berlin, Germany; 3 Klinik für Kinder- und Jugendmedizin, Pädiatrische Onkologie und Hämatologie, Universitätsklinikum Schleswig-Holstein, Campus Lübeck, Ratzeburger Allee 160, Haus A, 23538 Lübeck, Germany; 4 Department of Haematology, Oncology and Tumor Immunology, Charité-Universitätsmedizin Berlin, Corporate Member of Freie Universität Berlin, Humboldt-Universität zu Berlin, Berlin Institute of Health, Campus Virchow-Klinikum, Augustenburger Platz 1, Mittelallee 11, 13353 Berlin, Germany; 5 Institut für Medizinische Biometrie und Statistik (IMBS), Universität zu Lübeck, Universitätsklinikum Schleswig-Holstein, Campus Lübeck, Ratzeburger Allee 160, V24, 23562 Lübeck, Germany; 6 Pädiatrische Hämatologie/Onkologie, Zentrum für Kinder- und Jugendmedizin, Universitätsklinikum Bonn, Venusberg-Campus 1, 53127 Bonn, Germany; 7 IT Center for Clinical Research, Lübeck, Universität zu Lübeck, Haus 32, Ratzeburger Allee 160, 23562 Lübeck, Germany; 8 Division of Childhood Cancer Epidemiology/German Childhood Cancer Registry, Institute of Medical Biostatistics, Epidemiology and Informatics (IMBEI), University Medical Centre of the Johannes Gutenberg University Mainz, 55101 Mainz, Germany

**Keywords:** Health, Quality of life, Epidemiology, Childhood cancer survivors, Long-term follow-up, Late effects

## Abstract

**Purpose:**

Childhood cancer survivors (CCS) are at risk for increased morbidity and reduced quality of life associated with treatment-related late effects. In Germany, however, only a few of the more than 40,000 CCS registered in the German Childhood Cancer Registry (GCCR) currently benefit from adequate clinical long-term follow-up (LTFU) structures. To establish a comprehensive knowledge base on CCS’ long-term health in Germany, a database was developed in cooperation with the GCCR. Following a first evaluation phase at two German university centres, this database will be implemented more widely within Germany allowing longitudinal documentation of clinical LTFU data.

**Methods:**

The feasibility study cohort comprised 208 CCS aged 18 or older whose medical, mental and psychosocial health data were collected during routine LTFU or first clinic visits in adult care. CCS were enrolled from 04/2021 to 12/2022, and data entry was completed by 03/2023. Descriptive data analysis was conducted. All CCS were stratified into three risk groups (RG) based on their individual risk for developing late effects resulting from their respective diagnoses and treatments.

**Results:**

Chronic health conditions of various organ systems associated with late and long-term effects of cancer therapy affected CCS in all RG supporting the clinical relevance of risk-adapted LTFU. Enrolment into the database was feasible and broadly accepted amongst CCS.

**Conclusion:**

Implementation of a clinical follow-up care infrastructure and database in Germany will pave the way to collect clinically evaluated and regularly updated health data of potentially over 40,000 German CCS and facilitate future national and international cooperation.

**Supplementary Information:**

The online version contains supplementary material available at 10.1007/s00432-023-05145-8.

## Introduction

Long-term survival of childhood cancer has steadily increased during the last decades resulting in a growing community of childhood cancer survivors (CCS) worldwide (Robison and Hudson 2014). As many CCS face therapy-related chronic health conditions (“late effects”), adequate and risk-adapted clinical long-term follow-up (LTFU) care that aims at timely diagnosis and treatment of late effects is important to prevent subsequent morbidity and reduced health-related quality of life (Bhakta et al. 2017; Dixon et al. 2018). Most LTFU recommendations are based on experiences from large CCS cohorts that have been followed up regularly as part of regional or nation-wide CCS programmes (Kremer et al. 2013). At the beginning of this century, one of the largest CCS cohorts, the multi-centre North-American Childhood Cancer Survivor Study (CCSS), was established to facilitate research on long-term health outcomes in this patient group (Robison et al. 2002). It started out with a survey of over 20,000 CCS who had been diagnosed prior to the age of 21 between 1970 and 1986 and had survived for 5 years or more (Wang et al. 2022). The CCSS continues to assess various health issues in this expanding cohort of currently over 35,000 CCS. In addition, a clinical follow-up programme (SJLIFE) was set up, initially including over 9000 adult 10-year CCS originally treated at St. Jude Children’s Research Hospital, who periodically return to Memphis for comprehensive clinical evaluations (Hudson et al. 2011). Similar programmes have been set up in several European countries such as the Netherlands (Winther et al. 2015; Feijen et al. 2023).

In Germany, since 1980, childhood cancer-related data of all children and adolescents with cancer have been routinely collected by the German Childhood Cancer Registry (GCCR), embedded in the Division of Childhood Cancer Epidemiology (EpiKiK) at the University Medical Centre of Mainz of Johannes Gutenberg University. The GCCR-based cohort now exceeds 40,000 5-year CCS (Kaatsch et al. 2022). At least every 5 years, the GCCR carries out questionnaire-based follow-up on relapses and subsequent neoplasms as part of mandated duties (Kaatsch et al. 2022; Erdmann et al. 2020; Grabow et al. 2011; Langer et al. 2018). In addition, EpiKiK/GCCR-affiliated investigators have initiated and collaborated on numerous (inter)national studies on long-term health-related outcomes (Kaatsch et al. 2022; Byrne et al. 2022; van den Oever et al. 2023; Kaatsch et al. 2021; Aleshchenko et al. 2022; Botta et al. 2022).

Multidisciplinary LTFU clinics have been established at several university clinics in Germany recently (Gebauer et al. 2018), offering specialised and risk-adapted surveillance in CCS according to current guidelines (Gebauer et al. 2020). During CCS’ routine LTFU clinic visits, data on their current health status is routinely collected. We intend to document this clinically validated and prospectively assessed information comprehensively in a common database. These data can serve as a basis for future cooperation in national and international projects on chronic health conditions in CCS and facilitate joint analysis with CCS cohorts from other countries. We now present this clinical LTFU database including an analysis of the data collected during a first evaluation phase.

## Materials and methods

The database includes seven categories on CCS’ health and psychosocial status as well as on previous cancer disease(s) and corresponding treatment. The categories are based on common late effects addressed in LTFU guidelines (van Kalsbeek et al. 2021) and allow free text entry for the documentation of rare chronic health conditions. Subcategories include further specifications of diseases according to standard diagnostic procedures and, if applicable, to the International Classification of Diseases (ICD) (Harrison et al. 2021).

Following a pre-test in Luebeck in 2018 (Gebauer et al. 2018), the clinical LTFU database was modified for technical and pragmatic reasons before a first evaluation period. From April 2021 until December 2022, all CCS who survived ≥ 5 years after first cancer diagnosis and attended the LTFU clinics in Luebeck and Berlin were asked to provide their written informed consent for inclusion of their data in the database. This study was approved by the ethics committee of the University to Luebeck (registration number 180-87) and the ethics committee in Berlin waived this requirement.

Depending on the type of information, required data were entered into the database either once or repeatedly during each LTFU clinic visit. Risk-adapted LTFU in the participating clinics was carried out according to published guidelines (Gebauer et al. 2020) and served as the basis for the documentation of clinical data. At inclusion into the database, participants were asked to complete five validated self-report questionnaires and one questionnaire on their living situation via a study tablet to assess mental health, psychosocial burden and fatigue (Table 1). Information from the tablet was automatically transferred to the database following data protection guidelines. Psychologists or social workers specialised in LTFU assessed the psychosocial status of the CCS during the LTFU visit considering the answers to the questionnaires to identify if further support was required.

**Table 1:** Content of the clinical long-term follow-up (LTFU) database for German childhood cancer survivors

Category	Content	Frequency
**Cancer diagnosis and treatment information required for risk stratification**		
Initial cancer diagnosis^a^	Cancer entity Treatment details	Once
Relapse (initial cancer diagnosis)^a^	Time and localisation of relapse Treatment of relapse	Once for each relapse
Subsequent cancer^a^	Cancer entity Treatment details	Once for each subsequent cancer
Relapse of subsequent cancer^a^	Time and localisation of relapse Treatment of relapse	Once for each relapse of each subsequent cancer
**Health outcomes**		
Clinical examination and chronic diseases^a^	Results of physical examination Assessment of: Endocrine/metabolic diseases Cardiac diseases Pulmonary diseases Liver diseases Renal diseases Gastrointestinal disorders Immunodeficiency/splenic dysfunction Skin disorders Haematological diseases Hearing/visual impairment Substance abuse Medical history Vaccination record Medications Movement disorders (Neuro)psychiatric diseases Dental disorders Subsequent neoplasms (benign)	Every LTFU clinic visit
Family history/genetics^a^	Tumour predisposition syndrome Cardiovascular disease and cancer in first-degree relatives	Once and in case of change
Psychosocial history^a^	Content of consultation Need for further support/therapy	Every LTFU clinic visit
Living situation^b^	Marital status Education/employment Parenthood/ fertility Degree of disability	Every LTFU clinic visit (RG1: once and after 5 years)
PHQ-9-D (Gräfe et al. 2004)^b^	Depression severity	Once and after 5 years
GAD-7 (Spitzer et al. 2006)^b^	Generalised anxiety disorder	Once and after 5 years
NCCN distress-thermometer (Mehnert et al. 2006)^b^	Distress due to problems in 5 areas of life: practical, family, emotional, spiritual/religious, and physical problems	Every LTFU clinic visit (RG1: Once and after 5 years)
IES-R (Maercker and Schützwohl 1998)^b^	Post-traumatic stress disorder (PTSD) symptoms	Once and after 5 years
EORTC QLQ-C30 (Aaronson et al. 1993; Giesinger et al. 2020) and -FA12 (Weis et al. 2017)^b^	Quality of life in cancer patients and cancer-related fatigue	Once and after 5 years

*RG1* risk group 1 (for details see Risk group (RG) stratification within Materials and Methods), *PHQ-D* Patient Health Questionnaire—German version, includes PHQ-9-D (on depression), *GAD-7* Generalised Anxiety Disorder (on anxiety), *NCCN* National Comprehensive Cancer Network (US), *IES-R* impact of event-scale revised, *EORTC QLQ-C30* EORTC core quality of life of cancer patients, *EORTC QLQ-FA12* EORTC module cancer-related fatigue

^a^Completed from available clinical records at first clinical follow-up

^b^Completed by the patient via tablet

### Risk group (RG) stratification

A previously published definition of RG based on former risk stratification models (Frobisher et al. 2017) and adopted to the German health care system (Gebauer et al. 2020) was used to stratify CCS according to their risk for late effects. RG1 with a low risk for developing late effects comprises CCS who had only required surgical therapy [with exception of CCS with a central nervous system (CNS) tumour] as well as CCS of acute lymphatic leukaemia (ALL) or non-hereditary retinoblastoma who received chemotherapy only. CCS with intermediate risk for late effects (RG2) had received chemotherapy (excluding ALL and non-hereditary retinoblastoma) or, in case of a CNS tumour, had merely undergone surgical therapy. RG3 includes CCS at high(er) risk for late effects, such as after haematopoietic stem cell transplantation and/or irradiation.

### Statistical analysis

Statistical analyses were conducted using SPSS IBM Version 29. Descriptive statistics were carried out for CCS whose data were completed in the database until March 22, 2023. All continuous variables are presented as means and standard deviations (SD) and as median values and interquartile ranges (IQR). Comparisons according to RG and for numerical variables were made using Kruskal–Wallis test to compare means of metric variables, such as age and body mass index, for which data were not normally distributed, and chi-square-test for other non-metric variables. Generated asymptotic p values are of purely descriptive nature. Cancer diagnoses were grouped into main categories for analyses (see Table 2). Survivors of a malignancy in adulthood, who, considering the nature of their diagnoses, had required treatment in paediatric oncology (e.g. young adults with medulloblastoma), were included in the analyses.

**Table 2:** General characteristics of adult childhood cancer survivors enrolled into the database between 04/2021 and 12/2022 (*n* = 208)

	MD	Total
**Characteristics**		
**Age at time of inclusion**^a^ [years]	0	
Mean ± SD (range)		26.5 ± 10.3 (18.0–60.0)
Median (IQR)		21.6 (14.2)
**Sex** [*n*]	0	
Female		120 (57.7%)
Male		88 (42.3%)
**Risk group** [*n*]	0	
1—Low risk for late effects		40 (19.2%)
2—Intermediate risk for late effects		52 (25.0%)
3—High risk for late effects		116 (55.8%)
**Oncological diagnosis** [*n*]	0	
Leukaemia		76 (36.5%)
Lymphoma		55 (26.4%)
Central nervous system tumour		31 (13.9%)
Bone and soft-tissue tumour		20 (9.6%)
Embryonal tumour		12 (5.8%)
Others^b^		16 (7.7%)
**Age at time of diagnosis** [years]	1	
Mean ± SD (range)		10.0 ± 6.8 (0.0–34.7)
Median (IQR)		9.3 (10.1)
**Year of diagnosis** [*n*]	1^c^	
1961–1980		8 (3.9%)
1981–2000		54 (26.1%)
2001–2017		145 (70.0%)
**Time since diagnosis**^d^ [years]/[*n*]	1	
Mean ± SD (range)		16.4 ± 9.5 (5.0–57.7)
Median (IQR)		14.6 (12.2)
5 years		11 (5.3%)
6–10 years		60 (29.0%)
11–15 years		49 (23.7%)
> 15 years		87 (42.0%)
**Treatment details** [*n*/*N*]		
Surgical therapy, yes	73	73/135 (54.1%)
Chemotherapy, yes	23	173/185 (93.5%)
Irradiation, yes	0	85/208 (40.9%)
Mean dose [Gy]	11	31.69 ± 17.76 (12.00–72.00)
First haematopoietic stem cell transplantation, yes	0	19/208 (9.1%)
Autologous		8/19 (42.1%)
Allogenic		11/19 (57.9%)
Chronic graft-versus-host disease, yes	15	1/4 (25.0%)
Antibody therapy, yes	90	1/118 (0.8%)
Endocrine therapy, yes	92	0/116 (0.0%)
**First relapse**		
Yes [*n*]	0	32 (15.4%)
**Age at time of relapse diagnosis** [years]	0	
Mean ± SD (range)		11.1 ± 7.3 (1.4–27.5)
Median (IQR)		11.0 (11.4)
**Time since primary diagnosis** [years]	0	
Mean ± SD (range)		2.7 ± 2.6 (0.5–13.5)
Median (IQR)		1.8 (2.2)
Relapse occurred following primary therapy, yes	17	15/15 (100.0%)
** Relapse diagnosis**	1	
Relapse of primary oncological disease		28/31 (90.3%)
** Type of relapse diagnosis**	26	
Local		5/6 (83.3%)
Metastasised		1/6 (16.7%)
Disseminated		0
**Subsequent neoplasm**	0	
Yes [*n*]		12 (5.8%)
1 Subsequent neoplasm		9 (4.3%)
2 Subsequent neoplasms		2 (1.0%)
3 Subsequent neoplasms		1 (0.5%)
**Age at time of first subsequent neoplasm** [years]	0	
Mean ± SD (range)		35.5 ± 13.9 (10.5–58.7)
Median (IQR)		34.4 (16.8)
**Time since primary diagnosis to first subsequent neoplasm** [years]	0	
Mean ± SD (range)		22.3 ± 9.4 (7.3–37.3)
Median (IQR)		23.7 (15.4)
**Type of first subsequent neoplasm**	0	
Skin cancer		6/12 (50.0%)
Cancer of gender-specific organs		3 (25.0%)
Lymphoma		1 (8.3%)
Central nervous system tumour		1 (8.3%)
Small bowel cancer		1 (8.3%)
**Relapse of subsequent neoplasm**	1	3 (25.0%)

In case the day of cancer diagnosis was missing, it was replaced by the first day of the month. If the month was missing, it was set as January. Missing years were analysed as missing data.

Additional to describing general patient characteristics, we present the database by assessing data on health conditions in CCS at time of (first) database entry according to organ system and by RG. Late effects are also presented by the number of affected (organ) systems per patient in the respective RG. Further health-related information, e.g. family history, genetic tumour predisposition, cardiovascular risk profile as well as detailed information on mental health and psychosocial status, has also been documented in the database (see Table 1) and will be evaluated in a prospective fashion in the future observational studies and embedded trials. For this first evaluation, we decided to focus on somatic health conditions.

## Results

### Participant characteristics

Overall, 208 patients out of 212 agreed to participate in the study (response rate: 98.1%). Mean age of patients at enrolment into the study was 26.5 years (range 18.0–60.0), of which the majority were young adults aged 18–24 years (62.5%). On average, 16.4 years (range 5.0–57.7) had passed since cancer diagnosis. Detailed patient characteristics are presented in Table 2. CCS in our cohort were mainly diagnosed with leukaemia (36.5%) or lymphoma (26.4%) in childhood or adolescence, whilst 13.9% had previously suffered from a brain tumour (Table 2). According to RG stratification, 19.2% of participating patients had a low (RG1), 25.0% an intermediate (RG2) and 55.8% a high risk (RG3) for late effects following their cancer treatment (Table 2).

*MD* missing data, *SD* standard deviation, *IQR* interquartile range

^a^Referring to age at time of first clinical follow-up examination

^b^Others: extracranial germ cell tumour, neuroendocrine tumour, adrenal cortex tumour, colon cancer, Langerhans cell histiocytosis

^c^Despite missing year of diagnosis, this patient is still evaluable as part of the study as per the inclusion criteria considering their date of clinical/oncological examination was documented as 2016, presupposing cancer diagnosis prior to that (> 5 years since diagnosis)

^d^Timespan between dates of primary oncological diagnosis and first clinical follow-up examination. Percentages may not add up to 100% due to rounding; percentages of same absolute numbers might differ as only valid values were analysed per variable

### Health conditions in CCS according to RG and organ system

Chronic health conditions were documented in a considerable number of CCS (Table 3). Endocrinological disorders were found in half of all CCS documented in the database (113/208, 54.3%). For a third of our cohort (75/207, 36.2%), at least one cardiovascular health condition was documented, and about a fifth of CCS had a degree of disability (20/95, 21.1%).

**Table 3:** Health conditions in adult childhood cancer survivors documented from medical records, presented per organ system and risk group (RG)

	MD	RG1 (*n* = 40)	RG2 (*n* = 52)	RG3 (*n* = 116)	Total (= 208)	*p* value*
**Age at time of inclusion**^a^	0					
Mean ± SD (range)		19.5 ± 3.3 (18.0–37.1)	24.6 ± 8.9 (18.0–54.1)	29.8 ± 11.2 (18.0–60.0)	26.5 ± 10.3 (18.0–60.0)	< 0.001
Median (IQR)		18.7 (0.7)	20.8 (8.4)	25.1 (16.9)	21.6 (14.2)	
**Lifestyle variables**						
**Body mass index**^b^ [kg/m^2^]/[*n*]	4					
Mean ± SD (range)		23.31 ± 5.51 (15.02–36.44)	24.87 ± 6.70 (15.24–54.69)	23.77 ± 5.17 (13.81–49.25)	23.96 ± 5.65 (13.81–54.69)	0.402
Median (IQR)		21.27 (6.88)	22.73 (7.61)	22.94 (6.58)	22.50 (6.83)	
Underweight^b^		5 (12.8%)	4 (7.8%)	15 (13.2%)	24 (11.8%)	
Normal weight^b^		22 (56.4%)	28 (54.9%)	56 (49.1%)	106 (52.0%)	
Overweight^b^		7 (17.9%)	10 (19.6%)	30 (26.3%)	47 (23.0%)	
Obesity^b^		5 (12.8%)	9 (17.6%)	13 (11.4%)	27 (13.2%)	
**Substance use**^c^						
Nicotine	0					
Yes		1/7 (14.3%)	4/37 (10.8%)	13/98 (13.3%)	18/142 (12.7%)	0.894
No		6/7 (85.7%)	33/37 (89.2%)	81/98 (82.7%)	120/142 (84.5%)	0.894
Not examined		0 (0.0%)	0 (0.0%)	4/98 (4.1%)	4/142 (2.8%)	
Alcohol^d^	0					
Yes		4/7 (57.1%)	26/37 (70.3%)	61/98 (62.2%)	91/142 (64.1%)	0.742
No		3/7 (42.9%)	11/37 (29.7%)	33/98 (33.7%)	47/142 (33.1%)	0.742
Not examined		0 (0.0%)	0 (0.0%)	4/98 (4.1%)	4/142 (2.8%)	
**Complete vaccination status**^e^	3					
Yes		8 (20.0%)	26 (51.0%)	46 (40.4%)	80 (39.0%)	0.093
No		1 (2.5%)	2 (3.9%)	16 (14.0%)	19 (9.3%)	0.093
Unknown		13 (32.5%)	14 (27.5%)	34 (29.8%)	61 (29.8%)	
Not examined		18 (45.0%)	9 (17.6%)	18 (15.8%)	45 (22.0%)	
**Organ system affected by at least one health condition**, yes						
Endocrinological disorders	0	11 (27.5%)	22 (42.3%)	80 (69.0%)	113 (54.3%)	< 0.001
Cardiovascular disorders	1	9 (22.5%)	11 (21.2%)	55 (47.8%)	75 (36.2%)	0.007
Pulmonary diseases	0	4 (10.0%)	7 (13.5%)	14 (12.1%)	25 (12.0%)	0.879
Liver diseases	0	2 (7.5%)	4 (7.7%)	14 (12.1%)	21 (10.1%)	0.639
Kidney diseases	0	1 (2.5%)	6 (11.5%)	11 (9.5%)	18 (8.7%)	0.280
Gut disorders	1	5 (12.5%)	9 (17.3%)	32 (27.8%)	46 (22.2%)	0.071
Ear–nose–throat disorders	2	0 (0.0%)	6 (11.8%)	24 (20.7%)	30 (14.6%)	0.006
Eye disorders^f^	1	0 (0.0%)	2 (3.8%)	6 (5.2%)	8 (3.9%)	0.337
Dental status	1	2 (5.0%)	4 (7.7%)	25 (21.7%)	31 (15.0%)	0.068
Neurological disorders	0	1 (2.5%)	9 (17.3%)	17 (14.7%)	27 (13.0%)	0.077
Psychological disorders^g^	0	0 (0.0%)	2 (3.8%)	10 (8.6%)	12 (5.8%)	0.096
Recognised degree of disability	113	2 (5.7%)	6 (21.4%)	12 (37.5%)	20 (21.1%)	0.003
Subsequent neoplasm	0	1 (2.5%)	0 (0.0%)	11 (9.5%)	12 (5.8%)	0.035

*MD* missing data, *RG1* low risk, *RG2* intermediate risk, *RG3* high risk for late effects

*p values are of purely descriptive nature, calculation for metric means and “yes” and “no” answers, not included were “unknown” and “not examined”

^a^Referring to age at time of first clinical follow-up examination; SD: standard deviation; IQR: interquartile range

^b^BMI cut-offs defined as per the *World Health Organization *(World Health Organization 1995): Underweight: ≤ 18.5 kg/m^2^, normal weight: 18.6–24.9 kg/m^2^, overweight: 25–29.9 kg/m^2^, obesity: ≥30kg/m^2^

^c^These data were only available at the LTFU clinic in Luebeck

^d^Not presented is detailed break-down of frequency as specified in the database, e.g. “daily” or “< 1x/month”

^e^complete vaccination status defined as per the *German Standing Committee on Vaccination* (Ständige Impfkommission 2023)

^f^Only cataract included

^g^Only depression according to clinical assessment included (further psychosocial assessment took place in a separate appointment with a psychologist or social worker and via screening questionnaires); Percentages may not add up to 100% due to rounding; percentages of same absolute numbers might differ as only valid values were analysed per variable

**Fig. 1:**
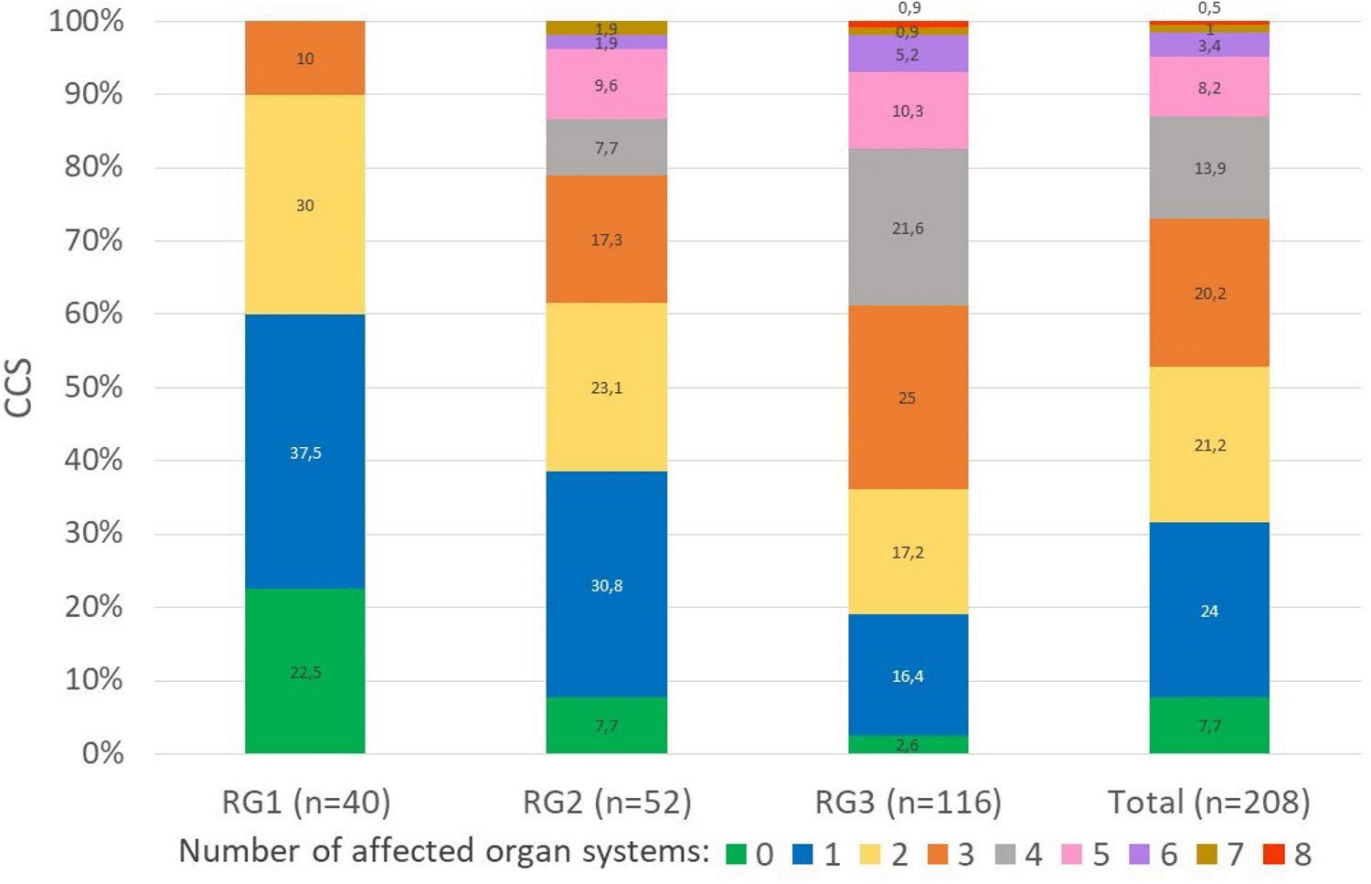
Rate of number of organ systems affected by health conditions documented in the database per childhood cancer survivor (CCS). RG1: low risk, RG2: intermediate risk, RG3: high risk for late effects. Order of legend labels/sections of respective bars: from bottom (“0 affected organ systems”) to top (“8 affected organ systems”)

Occurrence of health conditions in CCS differed amongst RG. In most categories, disorders consistently affected more CCS allocated to RG3 than CCS of RG1 or RG2 (Table 3). Overall, the number of affected organ systems increased with each RG (Fig. 1).

**Fig. 2:**
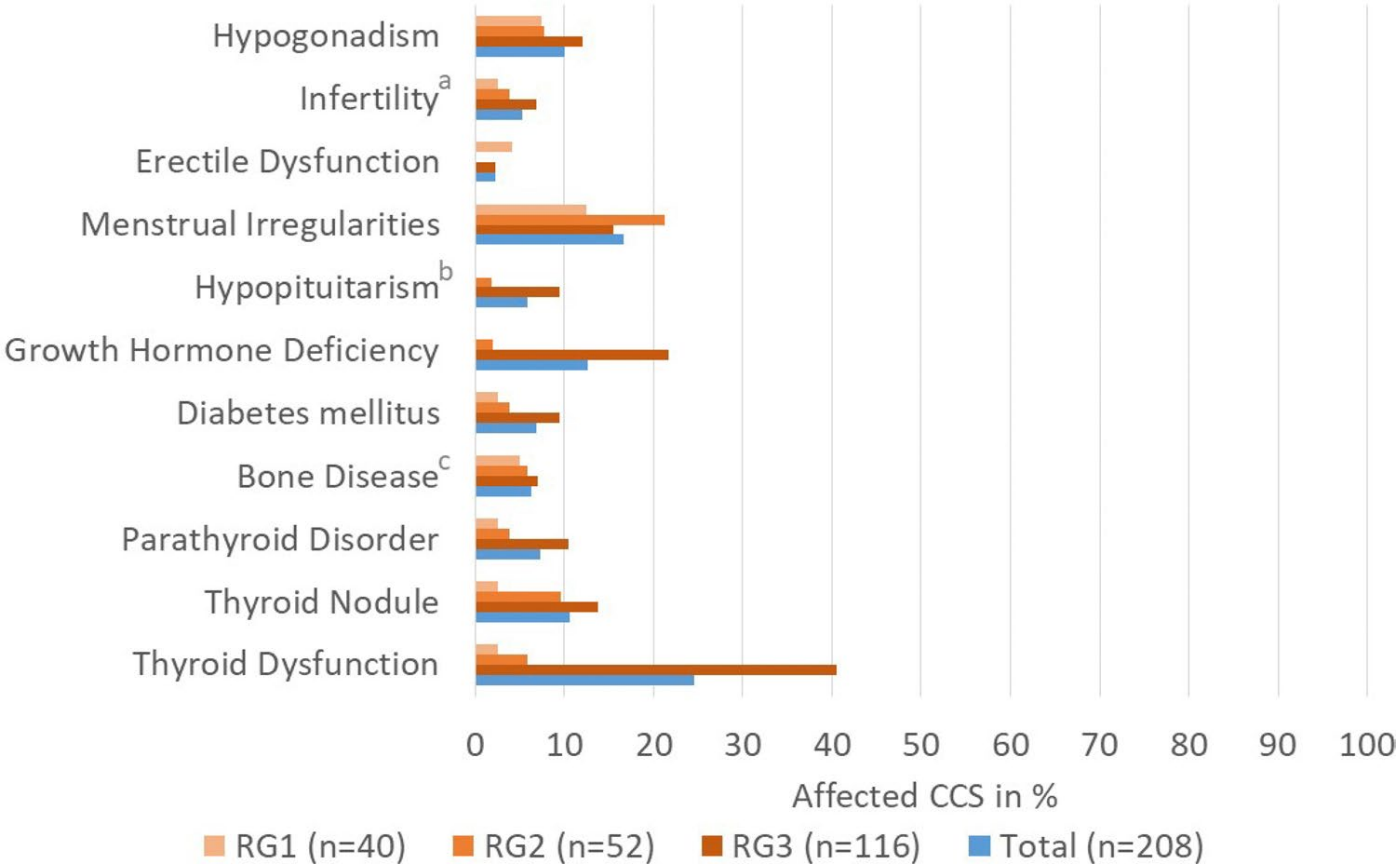
Endocrinological conditions in childhood cancer survivors (CCS) presented by risk group (RG). *RG1* low risk, *RG2* intermediate risk, *RG3* high risk for late effects. Order of legend labels of respective bars within conditions: from top (“RG1”) to bottom (“Total”). **a** only medically confirmed cases of infertility; **b** excluding cases with growth hormone deficiency only; **c** Bone status of survivors, for whom only vitamin D deficiency was selected, was regarded as unremarkable for statistical analysis of endocrinological conditions so as not to distort the results

Information from the database also allows a breakdown of single conditions within one organ system, e.g. specification of 11 different endocrinological conditions (Fig. 2).

**Fig. 3:**
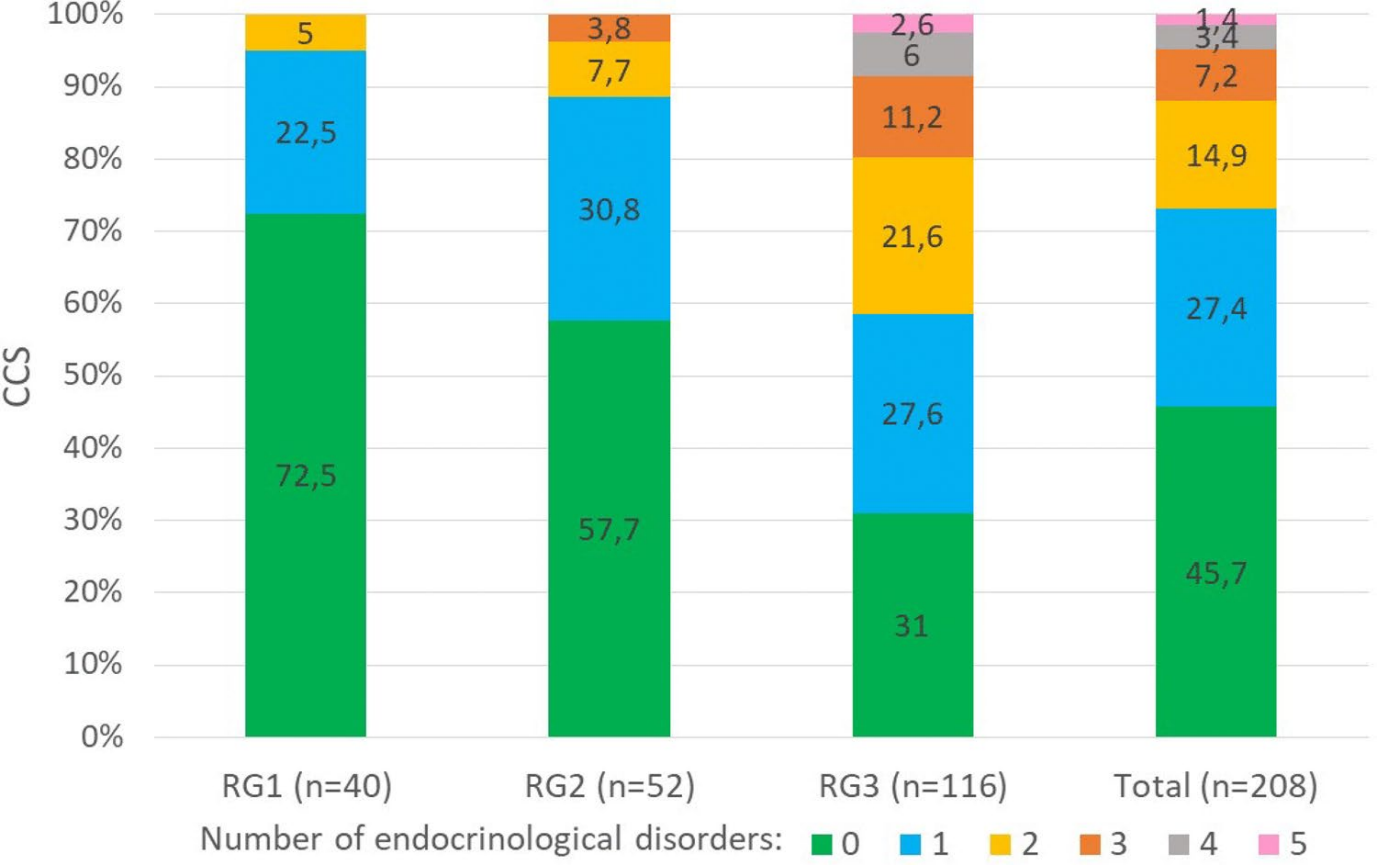
Rate of number of endocrinological disorders documented in the database per childhood cancer survivor (CCS). RG1: low risk, RG2: intermediate risk, RG3: high risk for late effects. Order of legend labels/sections of respective bars: from bottom (“0 endocrinological disorders”) to top (“5 endocrinological disorders”)

Number of endocrinological conditions ranged from 0 to 5 per CCS, with the majority having at least one specific condition (Fig. 3). Whilst two-third of CCS of RG1 had no endocrinological disorders and none had more than two, CCS of RG2 and RG3 suffered from up to three and five endocrinological conditions, respectively, with a lower proportion of CCS having none.

Furthermore, each subcategory was divided into several specifications providing detailed information on CCS’ health status. As an example, bone status comprised “unremarkable” (114/206, 55.3%), “vitamin D deficiency” (81/206, 39.3%), “osteopenia” (9/206, 4.4%), “osteoporosis” (3/206, 1.5%), “osteonecrosis” (3/206, 1.5%) and “not examined” (3/206, 1.5%). Percentages add up to more than 100% due to multiple selections (conditions) being possible per patient.

## Discussion

Long-term health consequences of cancer and its therapy affect most CCS, resulting in a reduced health-related quality of life and life expectancy in this growing cohort (Byrne et al. 2022; van Erp et al. 2021). Comprehensive risk-based care facilitates timely diagnosis and treatment of late effects and is recommended in numerous guidelines (Frobisher et al. 2017; Kremer et al. 2013). These recommendations are based on regularly updated, extensive analyses of CCS’ health status during LTFU. For some late effects, specific surveillance strategies are lacking as reliable data on occurrence and complications of some chronic health conditions are not available yet (Bowers et al. 2021). Recommendations in favour of or against surveillance modalities, however, require careful balancing of potential benefits and harms (Clement et al. 2018; Heinzel et al. 2022). Prospectively documented and evaluated late effects in nation-wide CCS cohorts can fill these knowledge gaps (Yeh et al. 2022) and serve as a basis for future joint projects. In several countries, national cohorts of CCS, who are regularly followed up by questionnaire surveys or clinically in specialised LTFU clinics, have been established (Winther et al. 2015). To provide comprehensive information on CCS’ health status in Germany, which goes beyond the current regular follow-up on subsequent neoplasms and relapses conducted by the GCCR, a database was created to support standardised prospective and longitudinal clinical data collection from routine LTFU assessments. Generally, acceptance of the database was successful in both participating clinics with almost all eligible CCS participating in this feasibility study.

Nationwide implementation of this database over the following years will provide clinically evaluated and regularly updated health data of potentially over 40,000 German CCS. These prospectively collected data can contribute to larger international analyses of CCS data, thus creating the basis for better tailored and individualised, risk-adapted LTFU recommendations.

According to the risk stratification approach (Frobisher et al. 2017) which was applied to our cohort, a considerable larger proportion of CCS allocated to RG3 (high risk for late effects) suffered from chronic health conditions than CCS of RG1 (low risk) and RG2 (intermediate risk). This difference is most pronounced for occurrence of endocrinological and cardiovascular disorders, as well as ear–nose–throat disorders, degree of disability and occurrence of subsequent neoplasms thus supporting the clinical relevance of the proposed risk stratification. Yet, it also needs to be considered that CCS in RG3 were generally older, so time since treatment was longest in this group. This limitation in this first data analysis will potentially be diminished once the cohort documented in the database exceeds a higher number of CCS. Number of health conditions with time increased in our cohort has also been previously described (Suh et al. 2020), emphasising the necessity of LTFU, potentially life-long. However, our findings also demonstrate an already high number of chronic health conditions in a cohort of young adult CCS, illustrated e.g. by a high occurrence of endocrinological disorders, which is in line with previous work and underlines the need for regular, life-long surveillance from the beginning of survivorship on Bhakta et al. (2017), Brignardello et al. (2013).

LTFU aims at holistic health assessment. The database correspondingly includes data on physical and mental health conditions, assessment of general health information, such as vaccination record, medical history and substance use as well as psychosocial and socioeconomic parameters. Analyses of this data provide an overview of the CCS’ characteristics as well as overall and in-depth assessment of their health status, e.g. alongside the documentation of organ systems affected by health conditions, also their type and extent are documented. Specific information on health care provision derived from data analyses can be directly transferred back into LTFU care and support its improvement by adapting to CCS’ needs.

To optimise data quality for future projects, careful consideration of possible imprecisions and errors in data assessment and documentation was conducted. In this context, some limitations were detected, e.g. some variables generating high numbers of missing data due to a missing “no” option. Interpretation of such missings as negation might distort results. For this first analysis within the feasibility study, we regard our approach as justifiable, seeing as findings in the affected variables would have likely been entered into the database had they been clear and available. For example, we argue that a relapse, had it occurred, would have been documented. For future use and implementation, a “no” option was added to these variables; for “infertility”, the variable “no clinical and laboratory evidence of infertility” will be added to the assessment. In addition, occurrence of hypogonadism could not be evaluated in women taking contraceptives if no further medical history was available. That is why our numbers might differ from expected prevalences from former studies (Mostoufi-Moab et al. 2016). Careful documentation of medical history and medications is essential to generate plausible data. A checklist will be forwarded to all participating LTFU centres to ensure complete data acquisition and to minimise missing data (Online Resource 1). Furthermore, to prevent systematic errors in data entry, a database manual containing background information on respective variables was provided and will be adapted for all future study centres. All identified limitations will be addressed in a database update to optimise future analyses.

## Conclusion and outlook

Risk-adapted clinical LTFU is crucial for CCS to ensure early detection of late effects of cancer treatment. However, in Germany, not all CCS benefit from such LTFU. Current structures need to be expanded to guarantee optimal health monitoring in all CCS. Our database enables a comprehensive assessment of health status in German CCS, including both general and detailed analyses of health aspects. Following a feasibility study in two LTFU clinics, data of further CCS that receive LTFU in German LTFU centres will be documented. Additionally, our database will be implemented in over ten centres in Germany as part of the prospective LE-Na trial (Evaluation and implementation of multidisciplinary, standardised, guideline-based long-term follow-up care for adult survivors of childhood cancer in Germany) which started in January 2023. During the study period of 5 years, over 5000 CCS who have not received standardised LTFU yet, will be invited to attend LTFU care in participating centres and asked for their data to be documented in the database. This comprehensive base on validated and prospectively collected CCS LTFU data will improve ongoing LTFU care and facilitate future national and international collaboration and research.

### Supplementary Information

Below is the link to the electronic supplementary material.

Supplementary file1 (PDF 45 KB)

## Data Availability

The anonymised data that support the findings of this study are available from the corresponding author upon reasonable request.

## References

[CR1] Aaronson NK (1993). The European Organization for Research and Treatment of Cancer QLQ-C30: a quality-of-life instrument for use in international clinical trials in oncology. J Natl Cancer Inst.

[CR2] Aleshchenko E (2022). Long-term care, care needs and wellbeing of individuals after cancer in childhood or adolescence (VersKiK): study protocol of a large scale multi-methods non-interventional study. BMC Health Serv Res.

[CR3] Bhakta N (2017). The cumulative burden of surviving childhood cancer: an initial report from the St Jude Lifetime Cohort Study (SJLIFE). Lancet.

[CR4] Botta L (2022). Long-term survival and cure fraction estimates for childhood cancer in Europe (EUROCARE-6): results from a population-based study. Lancet Oncol.

[CR5] Bowers DC (2021). Surveillance for subsequent neoplasms of the CNS for childhood, adolescent, and young adult cancer survivors: a systematic review and recommendations from the International Late Effects of Childhood Cancer Guideline Harmonization Group. Lancet Oncol.

[CR6] Brignardello E (2013). Endocrine health conditions in adult survivors of childhood cancer: the need for specialized adult-focused follow-up clinics. Eur J Endocrinol.

[CR7] Byrne J (2022). Impact of era of diagnosis on cause-specific late mortality among 77,423 five-year European survivors of childhood and adolescent cancer: the PanCareSurFup Consortium. Int J Cancer.

[CR8] Clement SC (2018). Balancing the benefits and harms of thyroid cancer surveillance in survivors of Childhood, adolescent and young adult cancer: recommendations from the international Late Effects of Childhood Cancer Guideline Harmonization Group in collaboration with the PanCareSurFup Consortium. Cancer Treat Rev.

[CR9] Dixon SB (2018). Factors influencing risk-based care of the childhood cancer survivor in the 21st century. CA Cancer J Clin.

[CR10] Erdmann F, Kaatsch P, Grabow D, Spix C (2020) German Childhood Cancer Registry - Annual Report 2019 (1980–2018). Institute of Medical Biostatistics, Epidemiology and Informatics (IMBEI) at the University Medical Center of the Johannes Gutenberg University Mainz

[CR11] Feijen EAM (2023). Clinical evaluation of late outcomes in Dutch childhood cancer survivors: methodology of the DCCSS LATER 2 study. Pediatr Blood Cancer.

[CR12] Frobisher C (2017). Risk stratification of childhood cancer survivors necessary for evidence-based clinical long-term follow-up. Br J Cancer.

[CR13] Gebauer J (2018). Multidisciplinary late effects clinics for childhood cancer survivors in Germany—a two-center study. Oncol Res Treat.

[CR14] Gebauer J (2020). Guidelines for long-term follow-up after childhood cancer: practical implications for the daily work. Oncol Res Treat.

[CR15] Giesinger JM (2020). Thresholds for clinical importance were established to improve interpretation of the EORTC QLQ-C30 in clinical practice and research. J Clin Epidemiol.

[CR16] Grabow D, Spix C, Blettner M, Kaatsch P (2011). Strategy for long-term surveillance at the German Childhood Cancer Registry—an update. Klin Padiatr.

[CR17] Gräfe K, Zipfel S, Herzog W, Löwe B (2004). Screening psychischer Störungen mit dem "Gesundheitsfragebogen für Patienten (PHQ-D)": Ergebnisse der deutschen Validierungsstudie. [Screening for psychiatric disorders with the Patient Health Questionnaire (PHQ). Results from the German validation study.]. Diagnostica.

[CR18] Harrison JE, Weber S, Jakob R, Chute CG (2021). ICD-11: an international classification of diseases for the twenty-first century. BMC Med Inform Decis Mak.

[CR19] Heinzel A, Müller D, van Santen HM, Clement SC, Schneider AB, Verburg FA (2022). The effect of surveillance for differentiated thyroid carcinoma in childhood cancer survivors on survival rates: a decision-tree-based analysis. Endocr Connect.

[CR20] Hudson MM (2011). Prospective medical assessment of adults surviving childhood cancer: study design, cohort characteristics, and feasibility of the St. Jude Lifetime Cohort study. Pediatr Blood Cancer.

[CR21] Kaatsch P, Byrne J, Grabow D (2021). Managing a Pan-European Consortium on late effects among long-term survivors of childhood and adolescent cancer—the PanCareLIFE Project. Int J Environ Res Public Health.

[CR22] Kaatsch P, Trübenbach C, Kaiser M, Erdmann F, Spix C, Grabow D (2022). The 41,000 long-term survivor cohort of the German Childhood Cancer Registry. Bundesgesundheitsblatt Gesundheitsforschung Gesundheitsschutz.

[CR23] Kremer LC (2013). A worldwide collaboration to harmonize guidelines for the long-term follow-up of childhood and young adult cancer survivors: a report from the International Late Effects of Childhood Cancer Guideline Harmonization Group. Pediatr Blood Cancer.

[CR24] Langer T (2018). Long-term follow-up in childhood cancer survivors—position paper 2018 of the working group "long-term follow-up" of the Society of Pediatric Oncology and Hematology (GPOH) on long-term surveillance, long-term follow-up and late effect evaluation in pediatric oncology patients. Klin Padiatr.

[CR25] Maercker A, Schützwohl M (1998). Erfassung von psychischen Belastungsfolgen: Die Impact of Event Skala-revidierte Version (IES-R). [Assessment of post-traumatic stress reactions: the Impact of Event Scale-Revised (IES-R).]. Diagnostica.

[CR26] Mehnert A, Müller D, Lehmann C, Koch U (2006). Die deutsche Version des NCCN distress-thermometers. Z Psychiatr Psychol Psychother.

[CR27] Mostoufi-Moab S (2016). Endocrine abnormalities in aging survivors of childhood cancer: a report from the childhood cancer survivor study. J Clin Oncol.

[CR28] Robison LL, Hudson MM (2014). Survivors of childhood and adolescent cancer: life-long risks and responsibilities. Nat Rev Cancer.

[CR29] Robison LL (2002). Study design and cohort characteristics of the Childhood Cancer Survivor Study: a multi-institutional collaborative project. Med Pediatr Oncol.

[CR30] Spitzer RL, Kroenke K, Williams JB, Löwe B (2006). A brief measure for assessing generalized anxiety disorder: the GAD-7. Arch Intern Med.

[CR31] Ständige Impfkommission (2023). Empfehlungen der Ständigen Impfkommission (STIKO) beim Robert Koch-Institut 2023. Epidemiologisches Bulletin.

[CR32] Suh E (2020). Late mortality and chronic health conditions in long-term survivors of early-adolescent and young adult cancers: a retrospective cohort analysis from the Childhood Cancer Survivor Study. Lancet Oncol.

[CR33] van den Oever SR (2023). Barriers and facilitators to implementation of the interoperable Survivorship Passport (SurPass) v2.0 in 6 European countries: a PanCareSurPass online survey study. J Cancer Surviv.

[CR34] van Erp LME (2021). Health-related quality of life in Dutch adult survivors of childhood cancer: a nation-wide cohort study. Eur J Cancer.

[CR35] van Kalsbeek RJ (2021). European PanCareFollowUp recommendations for surveillance of late effects of childhood, adolescent, and young adult cancer. Eur J Cancer.

[CR36] Wang Y (2022). Cohort profile: risk and risk factors for female breast cancer after treatment for childhood and adolescent cancer: an internationally pooled cohort. BMJ Open.

[CR37] Weis J (2017). International psychometric validation of an EORTC quality of life module measuring cancer related fatigue (EORTC QLQ-FA12). J Natl Cancer Inst.

[CR38] Winther JF (2015). Childhood cancer survivor cohorts in Europe. Acta Oncol.

[CR39] World Health Organization (1995). Physical status: the use and interpretation of anthropometry. Report of a WHO Expert Committee. World Health Organ Tech Rep Ser.

[CR40] Yeh JM (2022). Breast cancer screening among childhood cancer survivors treated without chest radiation: clinical benefits and cost-effectiveness. J Natl Cancer Inst.

