# Supplementary material for: Implementation of a clinical long-term follow-up database for adult childhood cancer survivors in Germany: a feasibility study at two specialised late effects clinics
Source: J Cancer Res Clin Oncol. 2023 Jul 18;149(14):12855–66. doi: 10.1007/s00432-023-05145-8 (PMC10587240; doi:10.1007/s00432-023-05145-8)
Supplement: Supplementary file 1 — Supplementary file1 (PDF 45 KB) [file 432_2023_5145_MOESM1_ESM.pdf]

Checklist for clinical long-term follow-up (LTFU) of childhood cancer survivors (CCS)

Article: Implementation of a clinical long-term follow-up database for adult childhood cancer survivors in Germany - A feasibility study at two specialised late effects clinics

Journal: Journal of Cancer Research and Clinical Oncology (J. Cancer Res. Clin. Oncol.)

Authors: Madelaine Sleimann, Magdalena Balcerek, Chirine Cytera, Franziska Richter, Anja Borgmann-Staudt, Bernhard Wörmann, Lea Louisa Kronziel, Gabriele Calaminus, Ann-Kristin Kock-Schoppenhauer, Desiree Grabow, Katja Baust, Anke Neumann, Thorsten Langer, Judith Gebauer

Corresponding Author: Dr. med. Judith Gebauer

Department of Internal Medicine I (Endocrinology and Diabetes)

University Hospital of Schleswig-Holstein

Ratzeburger Allee 160

23538 Luebeck

Germany

E-mail: judith.gebauer@uksh.de

Checklist for clinical long-term follow-up (LTFU) of childhood cancer survivors (CCS):

Initial cancer (including cancer treatment)	
Relapse? Second cancers?	
Chronic health conditions	
Current complaints	
Bowel movement	
Micturition / Nocturia	
Dyspnoea/Edema	
Changes in body weight	
Appetite	
Exercise / Sports	
Bone, Joint and Muscle complaints	
Sleep (quality)	
Tiredness/fatigue	
Depression	
Anxiety disorders	
Attention and concentration	
(Chronic) pain	
Loss of libido / erectile dysfunction	
Only women: menstrual cycle irregularities?	
(Unfullfilled) desire to have children/infertility?	
Cryopreservation?	
Eye / vision disorders	
Hearing disorders	
Neurological disorders / complaints	
Vaccination status	
Dental status	
Skin cancer screening	
Breast cancer screening	
Colon cancer screening	
Family history (cancer and cardiovascular diseases)	
Medication(s) including hormones and dietary supplements	
Smoking / Alcohol / Drugs	
Graduation / Profession	
Current occupation (hours/day)	
Marital status	

Physical examination:

Height:	Weight:	BMI:	Waist:
Blood pressure:	Pulse:		Hip:
	Rhythmic?		W/H-Ratio:

Skin/Hair/Nails:	Musculoskeletal:
Head/Neck/Thyroid:	Arms/Legs:
Heart/Lungs/Breast:	Neurological:
Abdomen:	Lymph nodes:

Further topics:
